# Efficacy and Safety of Glimepiride, Voglibose, and Metformin ER in Type 2 Diabetes: A Randomized, Active‐Controlled Study

**DOI:** 10.1111/1753-0407.70217

**Published:** 2026-04-14

**Authors:** Brij Mohan, S. Vasanth Kumar, Prakash Harishchandra Kurmi, Sandeep Kumar Gupta, Neelaveni Kudugunti, Bharat Das, Kartikeya Parmar, Alok Kanungo, Sanjay Saran, Preetam Ahirrao Kashinath, Balamurugan Ramanathan, Micky Patel, Vivek Vedprakash Arya, Raisa Shaikh, Sapan Behera, Piyush Patel, Dipak Patil, Lalit Lakhwani, Pravin Ghadge, Suyog Mehta, Sadhna Joglekar

**Affiliations:** ^1^ Brij Medical Centre Pvt Ltd Kanpur India; ^2^ Gandhi Hospital Secunderabad India; ^3^ Shivam Hospital Ahmedabad India; ^4^ M.V. Hospital and Research Centre Lucknow India; ^5^ Osmania General Hospital Hyderabad India; ^6^ Sparsh Hospital & Critical Care Private Limited Bhubaneswar India; ^7^ B.J. Medical College & Civil Hospital Ahmedabad India; ^8^ Dr. Alok Kanungo's Diabetic Center Bhubaneswar India; ^9^ SMS Medical College & Hospital Jaipur India; ^10^ Shree Siddhivinayak Maternity & Nursing Home Nasik India; ^11^ Kovai Diabetes Speciality Centre and Hospital Coimbatore India; ^12^ Lotus Multispeciality Hospital Ahmedabad India; ^13^ Smt. NHL Medical College and SVP Institute of Medical Sciences & Research Ahmedabad India; ^14^ Ex Sun Pharma Laboratories Limited Mumbai India; ^15^ Sun Pharma Laboratories Limited Mumbai India; ^16^ Ex Sun Pharmaceutical Industries Limited Mumbai India

**Keywords:** glimepiride, glycated hemoglobin, metformin, Type 2 diabetes, Voglibose

## Abstract

**Background:**

India ranks second in the global diabetes epidemic, with about 89 million individuals living with Type 2 diabetes mellitus (T2DM). We compared the efficacy and safety of fixed‐dose combination (FDC) of glimepiride, voglibose, and extended‐release metformin (GLIME+VOGLI+MET‐ER) with voglibose and metformin (VOGLI+MET) and glimepiride and metformin (GLIME+MET).

**Methods:**

A Phase IV, randomized, open‐label, active‐controlled study was performed in adult patients with T2DM poorly controlled with metformin. Patients received twice daily doses of GLIME+VOGLI+MET‐ER (1 + 0.2 + 500 mg: Trivolib 1, 2 + 0.2 + 500 mg: Trivolib 2); or VOGLI+MET (0.2 + 500 mg, 0.3 + 500 mg) or GLIME+MET (1 + 500 mg, 2 + 500 mg). The primary endpoint was the change in HbA1c from baseline.

**Results:**

Of the 458 patients screened, 399 were randomized (GLIME+VOGLI+MET‐ER [*n* = 133], VOGLI+MET (*n* = 135), and GLIME+MET [*n* = 131]). The mean baseline HbA1c was ~8.35% (~67.77 mmol/mol). All the treatments showed a significant reduction in HbA1c at Weeks 12 and 24. Mean change in % (mmol/mol) HbA1c was significantly more with GLIME+VOGLI+MET‐ER versus VOGLI+MET and GLIME+MET at Week 12 (−1.02 ± 0.60 [−11.2 ± 6.52] vs. −0.68 ± 0.64 [−7.49 ± 6.95], *p* < 0.001 and −0.88 ± 0.49 [−9.65 ± 5.34], *p* = 0.0154) and Week 24 (−1.57 ± 0.74 [−17.2 ± 8.14] vs. −1.11 ± 0.80 [−12.2 ± 8.79], *p* < 0.001 and −1.28 ± 0.60 [−14.0 ± 6.56], *p* = 0.0002). Overall, 49 adverse events (AEs) were reported in 32/399 (8.0%) patients. One patient each in GLIME+VOGLI+MET‐ER and VOGLI+MET groups had level 1 hypoglycemia requiring no management. No severe or serious AEs were reported.

**Conclusions:**

In adult patients with T2DM inadequately controlled on metformin monotherapy, GLIME+VOGLI+MET‐ER demonstrated superior HbA1c reduction compared with VOGLI+MET and GLIME+MET at Weeks 12 and 24. Study medications were safe and well‐tolerated.

**Trial Registration:** Clinical Trial Registry India—CTRI/2020/11/029080 [Registered on: 12/11/2020].

## Highlights

Triple therapy with a fixed‐dose combination of Glimepiride+Voglibose+Metformin‐extended release in patients with Type 2 diabetes mellitus (T2DM) demonstrated better glycemic control compared to dual therapies. A higher proportion of patients achieved target glycated hemoglobin levels (< 7.0%), with significant reductions in both fasting and postprandial glucose. This combination was well‐tolerated, with no increased risk of hypoglycemia, suggesting that Glimepiride+Voglibose+Metformin‐extended release could be an effective and safe option for patients with T2DM inadequately controlled with metformin.

## Graphical abstract

Triple therapy with a fixed‐dose combination of Glimepiride+Voglibose+Metformin‐extended release in patients with Type 2 diabetes mellitus demonstrated better glycemic control compared to dual therapies. This combination was well‐tolerated with no increased risk of hypoglycemia, suggesting that Glimepiride+Voglibose+Metformin‐extended release could be an effective and safe option for patients with Type 2 diabetes mellitus inadequately controlled with metformin.

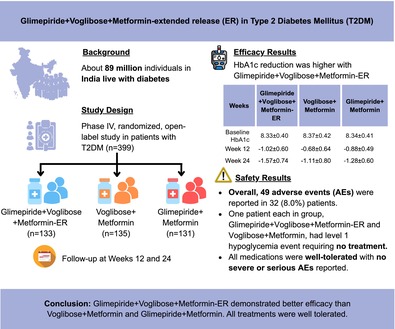

## 1 Introduction

Diabetes mellitus is a major public health concern with a global prevalence of 589 million as per the International Diabetes Federation 2025 report [1]. Among the countries most affected, India ranks second in the global diabetes epidemic. In 2024 alone, over 89 million Indian adults between the ages of 20 and 79 years were living with diabetes mellitus [2, 3]. Hence, there is an urgent need for more comprehensive public health strategies and innovative therapeutic approaches.

Type 2 diabetes mellitus (T2DM) is the predominant form of diabetes, accounting for more than 90% of all diabetes cases globally [4]. Due to the progressive nature of T2DM, patients with poor glycemic control require treatment with multiple anti‐diabetic drugs to achieve and maintain target glycated hemoglobin (HbA1c). Most patients are prescribed the traditional step‐up therapy consisting of initial metformin, followed by the addition of one oral anti‐diabetic drug. The majority of patients are prescribed triple‐drug therapies due to inadequate glycemic control with dual‐drug therapy. Numerous anti‐diabetic drugs are available today as monotherapy or fixed‐dose combinations (FDCs), and the availability of multiple therapeutic options is instrumental in helping reach the target HbA1c (a recognized strategy to prevent the complications of diabetes) [5].

The American Diabetes Association (ADA), consensus report by the ADA and European Association for the Study of Diabetes, the Indian Council of Medical Research (Diabetes guidelines 2018), and the Research Society for the Study of Diabetes in India‐Endocrine Society of India recommend combination therapy to achieve treatment goals. These guidelines advise a target HbA1c ≤ 7% (53.01 mmol/mol) for adequate glycemic control and minimizing the risk of complications [6, 7, 8, 9].

The major barrier in clinical practice is clinical inertia, resulting in a delay in optimizing therapy despite inadequate glycemic control. It may be difficult to achieve glycemic control by adding or initiating a single therapy when HbA1c levels are elevated. A double‐blind study in patients with T2DM uncontrolled with metformin monotherapy indicated that adding two drugs, that is, saxagliptin and dapagliflozin, was well tolerated and demonstrated better glycemic improvements as compared to the addition of one drug, that is, saxagliptin or dapagliflozin [6]. Combination therapies are based on the rationale of a multitargeted approach, and they also help in achieving and maintaining the desired glycemic targets. The advantages of FDCs include ease and convenience of administration, complementary mechanisms of action, the possibility of synergistic effects, and fewer side effects. These FDCs are also economically feasible and reduce pill burden and thereby provide improved treatment compliance, improved glycemic control, decreased incidence and severity of adverse events (AEs), and delayed need for insulin therapy [6, 10, 11]. However, limited clinical trial evidence is available that demonstrates the benefits of optimal use of available drugs, especially in combinations.

Several studies have investigated the efficacy of triple‐drug combination therapy in patients with T2DM. For instance, Abdul‐Ghani et al. reported that triple therapy using agents that enhance insulin sensitivity and preserve β‐cell function results in a sustained reduction in HbA1c compared to therapies that lower glucose levels without addressing the underlying metabolic abnormalities in patients with newly diagnosed T2DM [12]. Another study in drug‐naïve T2DM patients reported that initial triple therapy with metformin, sitagliptin, and empagliflozin resulted in achieving the glycemic target goal, which was maintained for 2 years, along with improved metabolic function and albuminuria, and no severe hypoglycemia [13]. A triple therapy of glimepiride, metformin, and voglibose was introduced due to their individual clinical benefits and synergistic action. An expert consensus regarding this triple‐drug therapy revealed that most healthcare providers believed that this combination may improve glycemic control and delay the occurrence of microvascular and cardiovascular complications [14]. Although a few studies have reported this triple‐drug therapy to be safe and effective in Indian patients with T2DM, none of the studies have evaluated its real‐world effectiveness with voglibose+metformin (VOGLI+MET) and glimepiride+metformin (GLIME+MET), both of which are approved by the CDSCO for use in India [15, 16, 17, 18, 19]. Therefore, we designed this study to evaluate the glycemic effectiveness of adding glimepiride, voglibose, or their combination to metformin in patients inadequately controlled on metformin monotherapy.

Importantly, emerging evidence suggests that early use of combination therapy with two or three agents at lower doses may be more effective than step‐wise escalation of a single agent to its maximum dose. This approach is based on simultaneously targeting multiple pathophysiological defects of T2DM rather than intensifying therapy directed at a single mechanism [12, 20]. Abdul‐Ghani et al. demonstrated that early combination therapy addressing core metabolic abnormalities of T2DM resulted in sustained glycemic control compared with conventional step‐wise treatment intensification [12].

The selected doses of glimepiride, voglibose, and metformin in the FDCs were based on approved clinical doses and prior evidence demonstrating efficacy with acceptable tolerability. Lower doses of individual agents were combined to leverage complementary mechanisms of action to achieve additive glycemic benefits while minimizing dose‐dependent AEs, particularly hypoglycemia and gastrointestinal intolerance, which are common barriers to treatment adherence [11, 14, 15, 16, 17, 18, 19, 21, 22, 23, 24].

Hence, we conducted this Phase IV study with a triple‐drug FDC in Indian patients having inadequate glycemic control with metformin monotherapy for ≥ 12 weeks, along with diet and exercise control. Given the increasing burden of T2DM in India, we hypothesize that the triple‐drug FDC of glimepiride (sulfonylurea), voglibose (*α*‐glucosidase inhibitor), and extended‐release (ER) metformin (biguanide), administered at lower complementary doses, would provide superior and sustained glycemic control, while maintaining an acceptable safety and tolerability profile in Indian patients with T2DM uncontrolled with metformin monotherapy.

## 2 Methods

### 2.1 Study Design and Participants

This was a prospective, multicenter, parallel‐group, open‐label, three‐arm, active‐controlled phase IV superiority study conducted across 14 centers in India. The study complied with the Good Clinical Practice guidelines, Declaration of Helsinki, New Drugs and Clinical Trials Rules 2019, and Indian Council of Medical Research guidelines 2017 [25, 26, 27]. The study protocol was approved by the regulatory authority, and the study was initiated after receiving approval from the institutional ethics committee at respective sites. Written informed consent was obtained from all participants. This trial was prospectively registered in the Clinical Trials Registry of India (CTRI/2020/11/029080).

Adult patients aged 18 to 65 years of either sex (men and nonpregnant, nonlactating women) with T2DM, who, along with diet and exercise control, were on a stable daily dose of metformin 1000 mg for ≥ 12 weeks prior to enrollment with an HbA1c ≥ 7.5% (≥ 58.48 mmol/mol) to ≤ 9% (≤ 75 mmol/mol) were included in this study. Patients with fasting blood glucose (FBG) > 270 mg/dL (> 14.99 mmol/L) at enrollment, PPBG (2‐h post meal) concentration ≤ 200 mg/dL (≤ 11.1 mmol/L) at screening, significant renal or hepatic impairment (estimated glomerular filtration rate < 45 mL/min/1.73 m^2^; aspartate transaminase and alanine transaminase > 3×ULN), history of acute or chronic metabolic acidosis, with heart failure, with inflammatory bowel disease or intestinal ulcers or chronic enteric diseases related to digestion and absorption, body mass index > 35 kg/m^2^ were excluded. Patients with a clinically significant condition, which in the opinion of the investigator would compromise the well‐being of the patient or interfere with the conduct of the study, were also excluded.

### 2.2 Study Procedure

After confirming eligibility and obtaining consent, patients were randomized to the treatment groups using a computer‐generated randomization sequence. Although the participants and investigators were aware of the assigned interventions after allocation, the use of centralized concealment minimized selection bias. The randomized patient took the study medication for 24 weeks (treatment period).

Patients were randomized in a 1:1:1 ratio to receive twice daily doses of GLIME+VOGLI+MET‐ER: 1 + 0.2 + 500 mg (Trivolib) 1 tablet manufactured by Sun Pharma or dual‐drug FDC tablet of VOGLI+MET (0.2 + 500 mg) or dual‐drug FDC tablet of GLIME+MET (1 + 500 mg) orally with food. Further dose uptitration was done at the end of Week 12 in patients with HbA1c > 7.5% (> 58.48 mmol/mol). The uptitrated patients received twice daily uptitrated doses of triple‐drug GLIME+VOGLI+MET‐ER: 2 + 0.2 + 500 mg (Trivolib 2 tablet) or dual‐drug FDC tablet of VOGLI+MET (0.3 + 500 mg) or dual‐drug FDC tablet of GLIME+MET (2 + 500 mg) till the end of Week 24. Patients with HbA1c ≤ 7.5% (≤ 58.48 mmol/mol) were not uptitrated and continued on the initial dose till the end of Week 24.

The use of anti‐diabetic drugs (other than study medications and rescue medications that were allowed) was prohibited during the study. Rescue medication included anti‐diabetic drugs except glucagon‐like peptide‐1 analogs, metformin, other sulfonylureas, or *α*‐glucosidase inhibitors or their combinations; choice of rescue medication other than these, as well as their dosage, was as per the investigator's discretion.

### 2.3 Study Outcomes

The primary efficacy endpoint was the mean change in HbA1c from baseline to Weeks 12 and 24 within each treatment group, with predefined comparisons between the triple‐therapy group (GLIME+VOGLI+MET‐ER) and each dual‐therapy group (VOGLI+MET and GLIME+MET).

The secondary endpoints were mean change in FBG, PPBG, and proportion of participants achieving HbA1c < 7.0% (< 53.01 mmol/mol) at the end of Weeks 12 and 24, and proportion of participants requiring rescue medications and hypoglycemia management. Safety endpoint included treatment‐emergent AEs (TEAEs) during the study. All the analyses were between the triple‐therapy group (GLIME+VOGLI+MET‐ER) and each dual‐therapy group (VOGLI+MET and GLIME+MET).

### 2.4 Harms

Harms were defined and graded according to the Common Terminology Criteria for Adverse Events (CTCAE) version 5.0 and coded using the Medical Dictionary for Regulatory Activities (MedDRA) version 27.0. Hypoglycemia episodes were defined as blood glucose levels ≤ 54 mg/dL and/or symptoms of hypoglycemia.

### 2.5 Discontinuation Criteria

The various criteria for discontinuation from the study included consent withdrawal, serious or intolerable AE, lack of adherence to the study procedure, major protocol deviation, study termination, two or more hypoglycemia events, and administration of the maximum labeled or tolerated dose of rescue medication for 4 weeks or more. Patients who met the rescue medication criteria but had known hypersensitivity to rescue medication, and were either considered inappropriate for use of insulin as the rescue medication or were unwilling to initiate insulin, were also discontinued from the study.

### 2.6 Statistical Analyses

The study required a sample size of 113 in each group (total *N* = 339). Assuming 80% power, a common standard deviation (SD) of 0.8, and a 5% two‐sided significance level to demonstrate clinical significance, a total sample size of up to 399 was required, considering an attrition rate of up to 15%.

The Mann–Whitney *U* test was used to check if there was a significant difference in glycemic parameters between the groups, and the Wilcoxon signed‐rank test was used to check if there was a significant difference from baseline to the end of Weeks 12 and 24. To analyze the proportion of patients achieving HbA1c < 7.0% at the end of Weeks 12 and 24, patients requiring hypoglycemia management, the chi‐square test was used to test if there was a significant difference between the two groups. A post hoc analysis of the proportion of patients requiring uptitration at the end of Week 12 was performed using the chi‐square test. No multiplicity adjustment in efficacy was considered. Last observation or baseline observation carry forward (LOCF/BOCF) was performed to impute missing efficacy data.

Assessments during the study were performed on the modified intention to treat (mITT), intention to treat (ITT), per protocol (PP), and safety population. ITT population included all patients randomized in the study; PP population included all randomized patients who completed the study as per the protocol without any major protocol deviation; mITT population included all randomized patients who administered at least one dose of assigned medication and returned for at least one postbaseline evaluation visit; safety population included all randomized patients who received at least one dose of study medication.

## 3 Results

### 3.1 Baseline Characteristics

Between September 30, 2021 and August 12, 2022, a total of 458 patients were screened. Of these, 59 patients were excluded due to screening failure, and 399 eligible patients were randomly assigned to receive a twice daily dose of either GLIME+VOGLI+MET‐ER tablet (*n* = 133), dual‐drug FDC tablet of VOGLI+MET (0.2 + 500 mg) (*n* = 135), or dual‐drug FDC tablet of GLIME+MET (1 + 500 mg) (*n* = 131).

**FIGURE 1:**
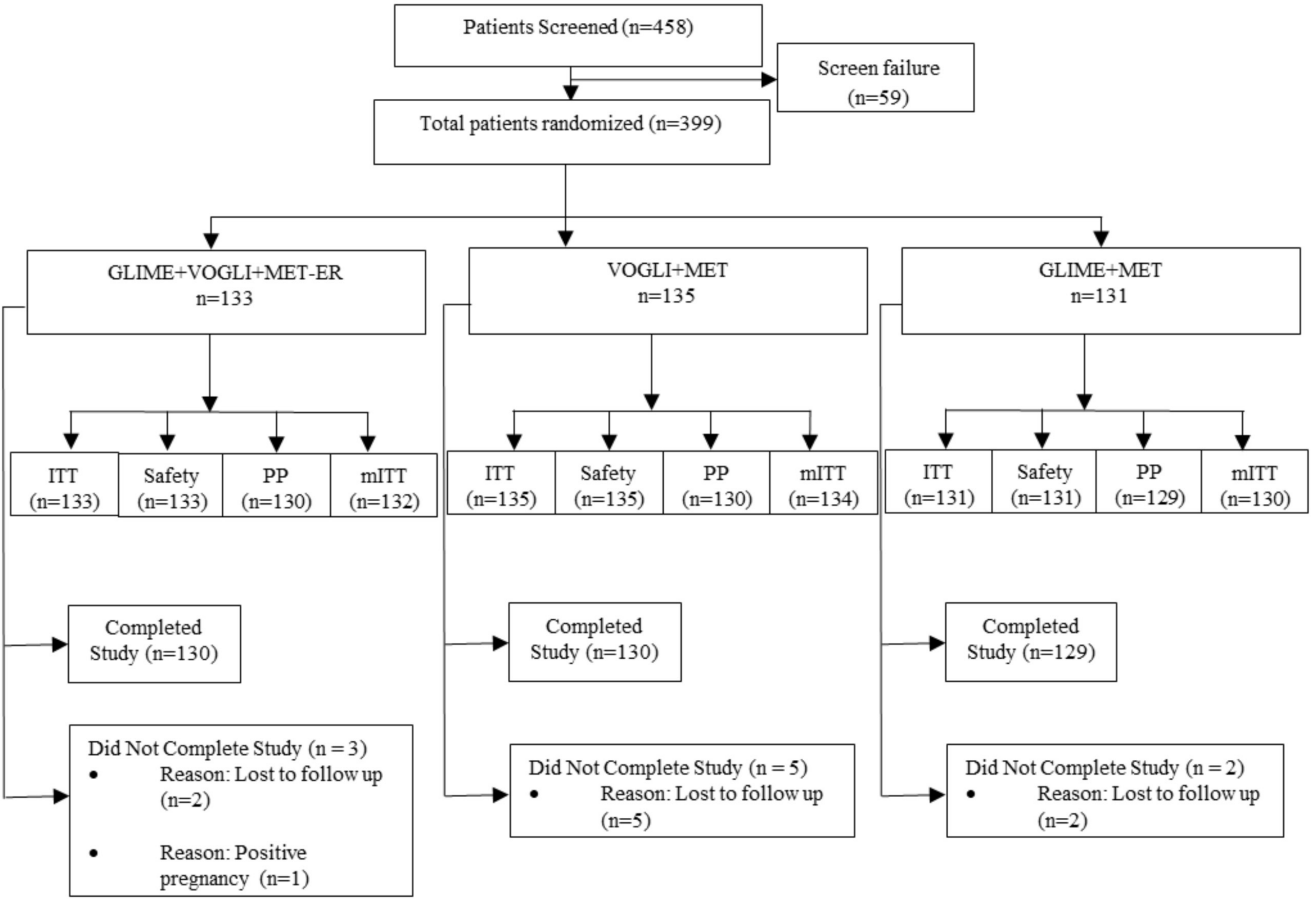
Patient disposition. ITT: intention to treat; mITT: modified intention to treat; *n*: number of patients; PP: per protocol. GLIME+VOGLI+MET‐ER: Glimepiride 1 mg, Voglibose 0.2 mg, and Extended‐Release Metformin 500 mg tablets; HbA1c: Glycated hemoglobin; VOGLI+MET: Voglibose 0.2 mg and Metformin 500 mg tablets; GLIME+MET: Glimepiride 1 mg and Metformin 500 mg tablets.

Figure 1 shows the outline of the trial; 389 patients completed the trial (GLIME+VOGLI+MET‐ER: *n* = 130; VOGLI+MET: *n* = 130; GLIME+MET: *n* = 129). Treatment compliance was 98.5%, 99.3%, and 99.2% in the GLIME+VOGLI+MET‐ER, VOGLI+MET, and GLIME+MET groups, respectively.

Table 1 shows the demographic and baseline characteristics of the participants in the safety population. Patients in each treatment group were comparable on age, sex, weight, and baseline HbA1c, FBG, and PPBG. Here, we report the efficacy results of the mITT population. The results of ITT and PP populations were similar to the mITT population. The safety results reported here are based on data from the safety population.

**TABLE 1:** Demographic and baseline characteristics of the participants.

Statistic summary (mean ± SD)	GLIME+VOGLI+MET‐ER (*n* = 133)	VOGLI+MET (*n* = 135)	GLIME+MET (*n* = 131)	GLIME+VOGLI+MET‐ER versus VOGLI+MET *p*‐value	GLIME+VOGLI+MET‐ER versus GLIME+MET *p*‐value
Sex *n* (%)				0.3218^a^	0.7878^a^
Male	76 (57.1%)	69 (51.1%)	77 (58.8%)		
Female	57 (42.9%)	66 (48.9%)	54 (41.2%)		
Age (years)	48.40 ± 9.29	48.93 ± 9.51	47.48 ± 9.53	0.6419^a^	0.4290^a^
Height (cm)	159.6 ± 7.91	159.8 ± 7.09	160.4 ± 6.58	0.8302^a^	0.3801^a^
Weight (kg)	68.34 ± 11.10	68.11 ± 9.50	68.59 ± 9.49	0.8567^a^	0.8451^a^
Body mass index (kg/m^2^)	26.74 ± 3.44	26.65 ± 3.20	26.62 ± 3.21	0.8100^a^	0.7683^a^
HbA1c % (mmol/mol)	8.33 ± 0.40 (67.59 ± 4.40)	8.37 ± 0.42 (67.94 ± 4.54)	8.34 ± 0.41 (67.60 ± 4.47)	0.4886^b^	0.9691^b^
FBG mg/dL (mmol/L)	177.8 ± 33.20 (9.87 ± 1.84)	179.2 ± 31.90 (9.94 ± 1.77)	178.1 ± 30.34 (9.88 ± 1.68)	0.6817^b^	0.9794^b^
PPBG mg/dL (mmol/L)	264.1 ± 46.65 (14.66 ± 2.59)	265.4 ± 39.01 (14.73 ± 2.16)	262.7 ± 39.44 (14.58 ± 2.19)	0.4118^b^	0.8455^b^

Abbreviations: FBG: fasting blood glucose; GLIME+MET: Glimepiride 1 mg and Metformin 500 mg tablets; GLIME+VOGLI+MET‐ER: Glimepiride 1 mg, Voglibose 0.2 mg, and Extended‐Release Metformin hydrochloride 500 mg tablets; HbA1c: Glycated hemoglobin; *n*: number of patients; PPBG: postprandial blood glucose; SD: standard deviation; VOGLI+MET: Voglibose 0.2 mg and Metformin 500 mg tablets.

^a^ *p*‐values are computed using two sample *T*‐test.

^b^ *p*‐values are computed using Mann–Whitney test.

### 3.2 Change in Glycated Hemoglobin

A statistically significant reduction (*p* < 0.0001) in %HbA1c was observed from baseline to Weeks 12 and 24 in each treatment group.

Mean ± SD change in % (mmol/mol) HbA1c from baseline to end of Week 12 was −1.02 ± 0.60 (−11.2 ± 6.52), −0.68 ± 0.64 (−7.49 ± 6.95), and −0.88 ± 0.49 (−9.65 ± 5.34) in the GLIME+VOGLI+MET‐ER, VOGLI+MET, and GLIME+MET groups, respectively. The HbA1c reduction was statistically significant in the GLIME+VOGLI+MET‐ER group compared to VOGLI+MET (*p* < 0.001) and GLIME+MET (*p* = 0.0154) groups at Week 12.

The %HbA1c improvement continued to increase, and the mean ± SD change in % (mmol/mol) HbA1c from baseline to end of Week 24 was −1.57 ± 0.74 (−17.2 ± 8.14), −1.11 ± 0.80 (−12.2 ± 8.79), and −1.28 ± 0.60 (−14.0 ± 6.56) in the GLIME+VOGLI+MET‐ER, VOGLI+MET, and GLIME+MET groups, respectively. A statistically significant reduction in %HbA1c was observed in the GLIME+VOGLI+MET‐ER group compared to VOGLI+MET (*p* < 0.001) and GLIME+MET (*p* = 0.0002) groups at Week 24.

**FIGURE 2:**
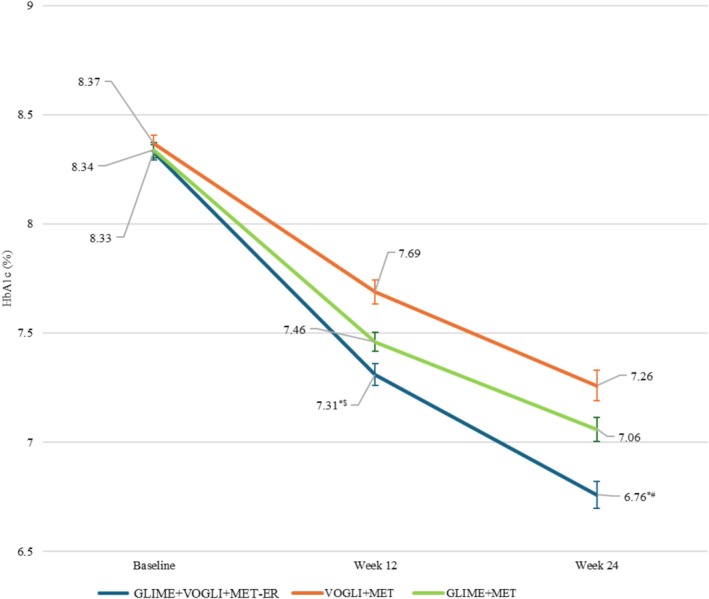
Change in glycated hemoglobin (%) from baseline to the end of Weeks 12 and 24. GLIME+MET: Glimepiride 1 mg and Metformin 500 mg tablets; GLIME+VOGLI+MET‐ER: Glimepiride 1 mg, Voglibose 0.2 mg, and Extended‐Release Metformin 500 mg tablets; HbA1c: Glycated hemoglobin; VOGLI+MET: Voglibose 0.2 mg and Metformin 500 mg tablets. *GLIME+VOGLI+MET‐ER group versus VOGLI+MET group at Weeks 12 and 24 (*p*‐value < 0.001 each); GLIME+VOGLI+MET‐ER group versus GLIME+MET group at Weeks 12 (^$^ *p*‐value = 0.0154) and 24 (^#^ *p*‐value = 0.0002).

The HbA1c values at baseline, Week 12, and Week 24 are indicated in Figure 2.

### 3.3 Change in Fasting Blood Glucose

A statistically significant reduction (*p* < 0.0001) in FBG was observed from baseline to Weeks 12 and 24 in each treatment group.

Mean ± SD change in FBG mg/dL (mmol/L) from baseline to end of Week 12 was −29.0 ± 30.50 (−1.61 ± 1.69), −30.4 ± 34.01 (−1.69 ± 1.89), and −30.9 ± 33.20 (−1.72 ± 1.84) in the GLIME+VOGLI+MET‐ER, VOGLI+MET, and GLIME+MET groups, respectively. Mean ± SD change in FBG mg/dL (mmol/L) from baseline to end of Week 24 was −54.4 ± 39.44 (−3.02 ± 2.19), −51.3 ± 40.97 (−2.85 ± 2.27), and −54.0 ± 35.21 (−3.00 ± 1.95) mg/dL in the GLIME+VOGLI+MET‐ER, VOGLI+MET, and GLIME+MET groups, respectively.

The results of GLIME+VOGLI+MET‐ER tablet were comparable with VOGLI+MET and GLIME+MET at Weeks 12 (GLIME+VOGLI+MET‐ER versus VOGLI+MET: *p* = 0.9719; GLIME+VOGLI+MET‐ER versus GLIME+MET: *p* = 0.8499) and 24 (GLIME+VOGLI+MET‐ER versus VOGLI+MET: *p* = 0.4937; GLIME+VOGLI+MET‐ER versus GLIME+MET: *p* = 0.7210).

### 3.4 Change in Postprandial Blood Glucose

A statistically significant reduction (*p* < 0.0001) in PPBG was observed from baseline to Weeks 12 and 24 in each treatment group.

Mean ± SD change in PPBG mg/dL (mmol/L) from baseline to end of Week 12 was −47.4 ± 46.09 (−2.63 ± 2.56), −41.8 ± 40.05 (−2.32 ± 2.22), and −44.2 ± 39.07 (−2.46 ± 2.17) in the GLIME+VOGLI+MET‐ER, VOGLI+MET, and GLIME+MET groups, respectively. Mean ± SD change in PPBG from baseline to end of Week 24 was −84.9 ± 57.49 (−4.71 ± 3.19), −76.4 ± 59.28 (−4.24 ± 3.29), and −81.3 ± 45.56 (−4.51 ± 2.53) mg/dL in the GLIME+VOGLI+MET‐ER, VOGLI+MET, and GLIME+MET groups, respectively.

At Week 12, a statistically significant improvement in PPBG was noted in the GLIME+VOGLI+MET‐ER group when compared to the VOGLI+MET group (*p* = 0.0367). The results were comparable between GLIME+VOGLI+MET‐ER and GLIME+MET groups (*p* = 0.4400). At Week 24, the results of GLIME+VOGLI+MET‐ER were comparable with VOGLI+MET and GLIME+MET groups (GLIME+VOGLI+MET‐ER versus VOGLI+MET: *p* = 0.4691; GLIME+VOGLI+MET‐ER versus GLIME+MET: *p* = 0.8742).

### 3.5 Proportion of Patients Achieving Glycated Hemoglobin < 7% (< 53.01 mmol/mol)

**FIGURE 3:**
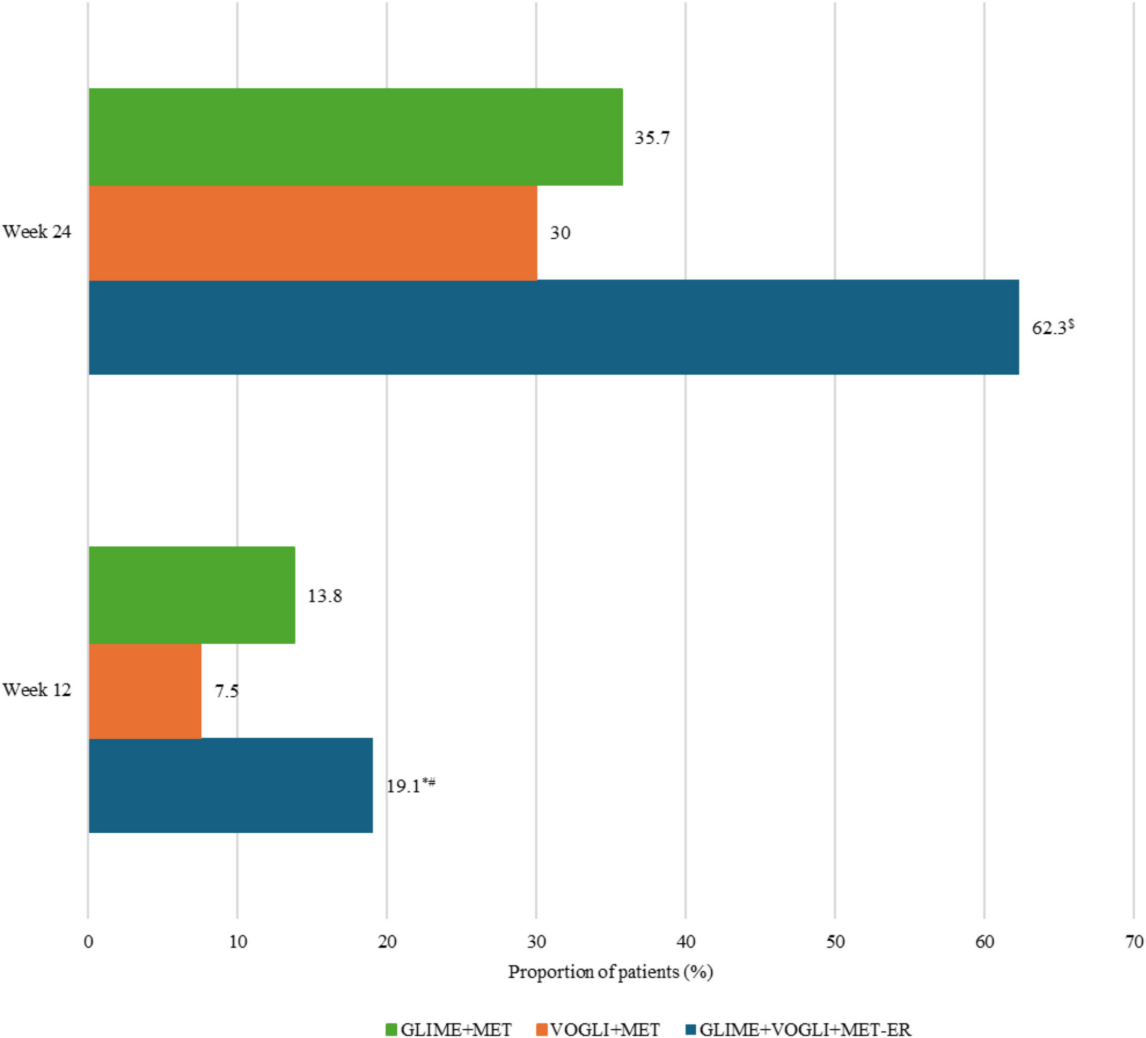
Proportion of patients achieving glycated hemoglobin < 7.0%. GLIME+MET: Glimepiride 1 mg and Metformin 500 mg tablets; GLIME+VOGLI+MET‐ER: Glimepiride 1 mg, Voglibose 0.2 mg, and Extended‐Release Metformin 500 mg tablets; VOGLI+MET: Voglibose 0.2 mg and Metformin 500 mg tablets. *p‐value = 0.0052 for GLIME+VOGLI+MET‐ER group versus VOGLI+MET group; ^#^ *p*‐value = 0.2541 for GLIME+VOGLI+MET‐ER group versus GLIME+MET group; ^$^ *p*‐value < 0.001 for GLIME+VOGLI+MET‐ER group versus VOGLI+MET and GLIME+MET groups.

As indicated in Figure 3, 25 (19.1%), 10 (7.5%), and 18 (13.8%) patients in the GLIME+VOGLI+MET‐ER, VOGLI+MET, and GLIME+MET groups, respectively, achieved HbA1c < 7.0% (< 53.01 mmol/mol) at the end of Week 12. A significantly greater number of patients achieved HbA1c < 7.0% (< 53.01 mmol/mol) in the GLIME+VOGLI+MET‐ER group compared to the VOGLI+MET group (*p* = 0.0052). Results were comparable between the GLIME+VOGLI+MET‐ER and GLIME+MET groups at Week 12 (*p* = 0.2541).

Similarly, 81 (62.3%), 39 (30.0%), and 46 (35.7%) patients in the GLIME+VOGLI+MET‐ER, VOGLI+MET, and GLIME+MET groups, respectively, achieved HbA1c < 7.0% (< 53.01 mmol/mol) at the end of Week 24. A significantly greater number of patients achieved HbA1c < 7.0% (< 53.01 mmol/mol) in the GLIME+VOGLI+MET‐ER group compared to VOGLI+MET and GLIME+MET groups (*p* < 0.001 each).

### 3.6 Proportion of Patients Requiring Uptitration

As indicated in Table 2, the proportion of patients requiring uptitration in the GLIME+VOGLI+MET‐ER group was significantly lower than both VOGLI+MET and GLIME+MET groups at the end of Week 12 (GLIME+VOGLI+MET‐ER vs. VOGLI+MET, 17.4% vs. 47.0%: *p* < 0.0001; GLIME+VOGLI+MET‐ER vs. GLIME+MET, 17.4% vs. 34.6%: *p* = 0.0015).

**TABLE 2:** Proportion of patients uptitrated at the end of Week 12.

	GLIME+VOGLI+MET‐ER (*n* = 132)	VOGLI+MET (*n* = 134)	GLIME+MET (*n* = 130)	GLIME+VOGLI+MET‐ER versus VOGLI+MET *p*‐value^a^	GLIME+VOGLI+MET‐ER versus GLIME+MET *p*‐value^a^
Proportion of patients uptitrated	23 (17.4%)	63 (47.0%)	45 (34.6%)	< 0.0001	0.0015

Abbreviations: GLIME+MET: Glimepiride 1 mg and Metformin 500 mg tablets; GLIME+VOGLI+MET‐ER: Glimepiride 1 mg, Voglibose 0.2 mg, and Extended‐Release Metformin hydrochloride 500 mg tablets; VOGLI+MET: Voglibose 0.2 mg and Metformin 500 mg tablets.

^a^ *p*‐values are computed using chi‐square test.

### 3.7 Proportion of Patients Requiring Rescue Medications

Only one (0.7%) patient in the VOGLI+MET group required rescue medication during the study. Teneligliptin was administered as rescue medication to this patient.

### 3.8 Proportion of Patients Requiring Hypoglycemia Management

Two patients had one asymptomatic hypoglycemia event each, out of which one (0.8%) patient (one event) was from the GLIME+VOGLI+MET‐ER group and one (0.7%) patient (one event) was from the VOGLI+MET group. Both hypoglycemia events were of level 1 severity, that is, measurable glucose concentration < 70 and ≥ 54 mg/dL. However, no patient required hypoglycemia management during the study.

### 3.9 Adverse Events

Overall, 49 TEAEs were reported in 32 (8.0%) patients out of 399 patients; out of these 49 TEAEs, 15 TEAEs were reported in 10 (7.5%) patients in the GLIME+VOGLI+MET‐ER group, 21 TEAEs were reported in 13 (9.6%) patients in the VOGLI+MET group, and 13 TEAEs were reported in 9 (6.9%) patients in the GLIME+MET group. No SAEs, deaths, severe or life‐threatening TEAEs were reported during the study. Out of 49 TEAEs, 48 TEAEs were mild in nature, and one TEAE was moderate in nature. The TEAE of moderate severity occurred in the VOGLI+MET group. Table 3 demonstrates the TEAEs reported in each group.

**TABLE 3:** Treatment‐emergent adverse events reported in each group.

System organ class preferred term	GLIME+VOGLI+MET‐ER (*n* = 133)		VOGLI+MET (*n* = 135)		GLIME+MET (*n* = 131)		Overall (*n* = 399)	
	Patients *n* (%)	Events	Patients *n* (%)	Events	Patients *n* (%)	Events	Patients *n* (%)	Events
Total TEAEs	10 (7.5%)	15	13 (9.6%)	21	9 (6.9%)	13	32 (8.0%)	49
Ear and labyrinth disorders	2 (1.5%)	2	1 (0.7%)	1	0	0	3 (0.8%)	3
Vertigo	2 (1.5%)	2	1 (0.7%)	1	0	0	3 (0.8%)	3
Gastrointestinal disorder	3 (2.3%)	3	7 (5.2%)	9	6 (4.6%)	6	16 (4.0%)	18
Abdominal pain upper	0	0	0	0	1 (0.8%)	1	1 (0.3%)	1
Constipation	0	0	1 (0.7%)	1	0	0	1 (0.3%)	1
Diarrhea	1 (0.8%)	1	2 (1.5%)	3	1 (0.8%)	1	4 (1.0%)	5
Hyperchlorhydria	0	0	1 (0.7%)	1	0	0	1 (0.3%)	1
Mouth ulceration	1 (0.8%)	1	1 (0.7%)	1	0	0	2 (0.5%)	2
Nausea	1 (0.8%)	1	2 (1.5%)	2	1 (0.8%)	1	4 (1.0%)	4
Vomiting	0	0	1 (0.7%)	1	3 (2.3%)	3	4 (1.0%)	4
General disorders and administration site conditions	1 (0.8%)	1	2 (1.5%)	2	2 (1.5%)	2	5 (1.3%)	5
Asthenia	0	0	0	0	1 (0.8%)	1	1 (0.3%)	1
Pain	0	0	0	0	1 (0.8%)	1	1 (0.3%)	1
Pyrexia	1 (0.8%)	1	2 (1.5%)	2	0	0	3 (0.8%)	3
Infections and infestations	1 (0.8%)	1	0	0	0	0	1 (0.3%)	1
Nasopharyngitis	1 (0.8%)	1	0	0	0	0	1 (0.3%)	1
Metabolism and nutrition disorders	1 (0.8%)	1	2 (1.5%)	2	0	0	3 (0.8%)	3
Hyperglycemia	0	0	1 (0.7%)	1	0	0	1 (0.3%)	1
Hypoglycemia	1 (0.8%)	1	1 (0.7%)	1	0	0	2 (0.5%)	2
Musculoskeletal and connective tissue disorders	0	0	0	0	1 (0.8%)	2	1 (0.3%)	2
Back pain	0	0	0	0	1 (0.8%)	2	1 (0.3%)	2
Nervous system disorders	2 (1.5%)	2	6 (4.4%)	7	3 (2.3%)	3	11 (2.8%)	12
Dizziness	0	0	1 (0.7%)	1	0	0	1 (0.3%)	1
Headache	2 (1.5%)	2	4 (3.0%)	4	2 (1.5%)	2	8 (2.0%)	8
Lethargy	0	0	1 (0.7%)	1	0	0	1 (0.3%)	1
Muscular weakness	0	0	1 (0.7%)	1	0	0	1 (0.3%)	1
Polyneuropathy	0	0	0	0	1 (0.8%)	1	1 (0.3%)	1
Respiratory, thoracic, and mediastinal disorders	4 (3.0%)	5	0	0	0	0	4 (1.0%)	5
Cough	3 (2.3%)	3	0	0	0	0	3 (0.8%)	3
Rhinorrhea	1 (0.8%)	1	0	0	0	0	1 (0.3%)	1
Sneezing	1 (0.8%)	1	0	0	0	0	1 (0.3%)	1

Abbreviations: GLIME+MET: Glimepiride 1 mg and Metformin 500 mg Tablets; GLIME+VOGLI+MET‐ER: Glimepiride 1 mg, Voglibose 0.2 mg, and Extended‐Release Metformin hydrochloride 500 mg tablets; *n*: number of patients; TEAEs: treatment‐emergent adverse events; VOGLI+MET: Voglibose 0.2 mg and Metformin 500 mg Tablets.

TEAEs neither led to a dose change nor to permanent discontinuation of the patient from the study. All TEAEs in this study were reported as “unlikely” related to study medications by the investigator and were resolved before the study completion.

## 4 Discussion

This phase IV, randomized, real‐world study demonstrates that in adult patients with T2DM inadequately controlled on metformin monotherapy, early treatment intensification with GLIME+VOGLI+MET‐ER provides superior and sustained glycemic control without additional safety concerns as compared to dual‐drug FDCs. Achievement of target HbA1c levels by Week 24 and the sustained glycemic response observed over 24 weeks supports the clinical applicability of this rational, lower‐dose triple‐drug strategy in routine practice, where long‐term glycemic durability and treatment tolerability remain major therapeutic challenges.

The findings are supported by previous studies demonstrating the efficacy and safety of glimepiride, voglibose, and metformin in various combinations for the management of T2DM. A prospective, parallel‐group, open‐label, active‐controlled study by Murti et al. demonstrated the change in HbA1c, FBG, and PPBG without (group A) and with (group B) the addition of voglibose to glimepiride and metformin therapy in the management of patients with T2DM. Groups A and B had significant reductions in mean FBG, PPBG, and HbA1c levels after 3 months. However, group B demonstrated a significant benefit in controlling FBG, PPBG, and HbA1c levels as compared to group A. These findings highlight that the addition of voglibose to the dual therapy of glimepiride and metformin can demonstrate better results when compared to dual therapy [28].

Further supporting evidence in a retrospective, observational, multicentric study by Bantwal et al., which included a total of 2650 adult patients of either sex with T2DM who had received treatment with different strengths of FDC of glimepiride, voglibose, and metformin with or without another anti‐diabetic agent. The mean HbA1c reduction was significant after the treatment (1.45%; *p* < 0.001). Overall results demonstrated that triple FDC of glimepiride, voglibose, and metformin was well tolerated and efficacious with improved compliance in Indian patients with T2DM [29]. Thus, the results of our study are in line with the published research.

Additionally, an expert consensus report by Das et al. physicians strongly opined that targeting postprandial hyperglycemia may delay the risk of cardiovascular disease and complications. Further, they recommended that early use of a triple‐drug combination of glimepiride, voglibose, and metformin improves glycemic control. This combination may also delay the risk of microvascular and macrovascular complications and delay the requirement of insulin. Additionally, they also stated that it is cost‐effective in Indian settings [14]. Taken together, these findings underscore the clinical rationale and real‐world evidence of using a triple‐drug FDC (GLIME+VOGLI+MET‐ER) in patients with inadequately controlled T2DM. The ability of the combination to target both FBG and PPBG, a favorable safety profile, and improved compliance due to reduced pill burden with FDC make it a promising therapeutic approach in routine clinical practice.

Although this study reported beneficial outcomes, there were a few limitations. The inclusion criteria restricted the baseline HbA1c to ≤ 9.0% and FBG ≤ 270 mg/dL, which limits the generalizability of the study findings. This study provides unique clinical evidence of the use of GLIME+VOGLI+MET‐ER with baseline HbA1c levels up to 9%, which may provide faster glycemic control. Moreover, the lack of this symptom‐based stratification may have introduced some heterogeneity in baseline disease severity. Additionally, this was an open‐label study, which could potentially introduce bias. However, randomization with centralized allocation concealment eliminated the selection bias, and the primary efficacy endpoint (HbA1c) was assessed objectively, thereby eliminating assessment bias. Although the recommended daily dose of GLIME+VOGLI+MET‐ER is twice daily with or without additional voglibose (based on the investigator's discretion), our study did not permit additional voglibose to avoid skewed data. While this improves the internal validity of our study, this may not completely reflect the real‐world dosing flexibility. The study permitted the use of other rescue medications during the study to ensure patient safety, and patients with uncontrolled blood glucose levels were discontinued from the study, which may have influenced the overall outcome measures. Despite these limitations, our study provides robust evidence supporting the efficacy and safety of GLIME+VOGLI+MET‐ER in patients with T2DM inadequately controlled on metformin monotherapy.

## 5 Conclusion

This study demonstrated that treatment with GLIME+VOGLI+MET‐ER resulted in a greater reduction in HbA1c compared to dual combination therapies in adult patients with T2DM inadequately controlled on metformin monotherapy. The glycemic benefits were sustained through 24 weeks. Additionally, a significantly higher proportion of patients achieved the glycemic target of HbA1c < 7.0% (53.01 mmol/mol) with GLIME+VOGLI+MET‐ER. Moreover, a significant reduction in FBG and PPBG was observed with this drug at Weeks 12 and 24, without any increased risk of hypoglycemia. All three treatments were generally well‐tolerated, with the majority of AEs reported as mild. Hence, GLIME+VOGLI+MET‐ER is a promising, effective, safe, and convenient therapeutic option for patients with inadequate glycemic control on metformin monotherapy.

## Author Contributions

Brij Mohan, S. Vasanth Kumar, Prakash Harishchandra Kurmi, Sandeep Kumar Gupta, Neelaveni Kudugunti, Bharat Das, Kartikeya Parmar, Alok Kanungo, Sanjay Saran, Preetam Ahirrao Kashinath, Balamurugan Ramanathan, Micky Patel, and Vivek Vedprakash Arya contributed to the conduct of the study, patient recruitment, and data acquisition at their respective sites, and reviewed the manuscript for important intellectual content. Sapan Behera, Piyush Patel, Lalit Lakhwani, and Suyog Mehta contributed to the study conception. Raisa Shaikh contributed to data analysis and interpretation, literature review, and drafting of the manuscript. Sapan Behera, Piyush Patel, and Dipak Patil contributed to data analysis and interpretation, literature review, and critical revision for intellectual content. Pravin Ghadge, Lalit Lakhwani, and Suyog Mehta contributed to overall study supervision, data interpretation, and critical review of the manuscript. Sadhna Joglekar contributed to the study design and critical review of the manuscript. All authors meet the International Committee of Medical Journal Editors (ICMJE) authorship criteria and contributed to final approval of the version to be published and agree to be accountable for all aspects of the work and ensure that questions related to the accuracy or integrity of any part of the work are appropriately investigated and resolved.

## Funding

The study was funded by the Sun Pharma Laboratories Limited.

## Ethics Statement

The study was initiated after receiving approval from the institutional ethics committee at respective sites.

## Consent

Written informed consent was obtained from all participants prior to participation in the study.

## Conflicts of Interest

Authors Brij Mohan, S. Vasanth Kumar, Prakash Harishchandra Kurmi, Sandeep Kumar Gupta, Neelaveni Kudugunti, Bharat Das, Kartikeya Parmar, Alok Kanungo, Sanjay Saran, Preetam Ahirrao Kashinath, Balamurugan Ramanathan, Micky Patel, and Vivek Vedprakash Arya were the study investigators, and they received grants for conducting the study at their respective sites and declare no conflicts of interest. Dipak Patil, Pravin Ghadge, and Suyog Mehta are full‐time employees of Sun Pharma Laboratories Limited. Raisa Shaikh, Sapan Behera, Piyush Patel, and Lalit Lakhwani were affiliated with Sun Pharma Laboratories Limited during the conduct of the study. Sadhna Joglekar was affiliated with Sun Pharmaceutical Industries Limited during the conduct of the study; she is currently employed by Novartis. The views expressed in the article are the authors' views and do not necessarily reflect the views or official position of Novartis.

## Data Availability

The data that support the findings of this study are available from the corresponding author upon reasonable request.
